# Molecular insights into salt stress response in perennial ryegrass (*Lolium perenne* L.): gene expression and growth performance assessment

**DOI:** 10.3389/fpls.2025.1741635

**Published:** 2026-01-05

**Authors:** Gözde Hafize Yıldırım, Seda Mesci, Şeyma Şengür, Muhammad Tanveer Altaf

**Affiliations:** 1 Faculty of Agriculture, Department of FieldCrops, Recep Tayyip Erdoğan University, Rize, Türkiye; 2 Project Coordination and Guidance Office, Rectorate, Hitit University, Çorum, Türkiye; 3 Food Safety, Agricultural Application and Research Center, Hitit University, Çorum, Türkiye; 4 Faculty of Agriculture, Department of Landscape Architecture, Ordu University, Ordu, Türkiye

**Keywords:** glutathione reductase, phytochelatin synthase, ryegrass, salt stress, stress-responsive genes

## Abstract

Perennial ryegrass (*Lolium perenne* L.) is a key perennial species with significant agricultural and ecological importance. Salt stress adversely affects plant growth by inducing oxidative stress, reducing biomass accumulation, and impairing physiological functions. In this study, calcium chloride, magnesium chloride, magnesium sulfate, and sodium sulfate treatments were applied to evaluate their effects on salinity-induced molecular and physiological responses. The effects of these treatments on the expression of salt stress–responsive genes Ascobate Peroxidase (APX), Glutathione Reductase (GR), Heavy Metal ATPase (HMA), and Phytochelatin Synthase (PCS) were analyzed using quantitative real-time PCR (qRT-PCR). In addition, agronomic traits including seedling length, fresh and dry weight, plant water content, and dry matter ratio were evaluated. Higher salinity increased stress-related gene expression, but this was not enough to maintain growth or water retention. In contrast, mild to moderate salt stress resulted in more balanced gene expression, reduced physiological damage, and improved plant development. These findings provide insights into the molecular and physiological responses of perennial ryegrass to different salt sources and may support future research on improving salinity tolerance in forage species.

## 1 Introduction

Perennial ryegrass (*Lolium perenne* L.) is a widely cultivated cool-season grass valued for its high adaptability, forage quality, and resilience, and is commonly used in temperate regions for forage production and turf systems (Miao et al., 2022). Among the environmental stress factors intensified by climate change, soil salinity is one of the most critical constraints limiting plant growth and productivity. Commercial cultivars of *L. perenne* generally exhibit only moderate salt tolerance, which restricts their performance under saline soil conditions (Li et al., 2023; Liu et al., 2019). Therefore, elucidating the physiological and molecular mechanisms underlying salt tolerance in *L. perenne* is essential for improving its sustainability and productivity under suboptimal environmental conditions.

The impact of salinity on plant performance varies depending on the chemical composition of salts in the soil. While the effects of sodium chloride (NaCl) are well documented, other salts such as CaCl_2_, MgCl_2_, MgSO_4_, and Na_2_SO_4_ can differentially influence growth, osmotic balance, and oxidative stress, depending on species, genotype, ionic composition, and exposure duration (Tushar et al., 2012); however, other salts such as calcium chloride (CaCl_2_), magnesium chloride (MgCl_2_), magnesium sulfate (MgSO_4_), and sodium sulfate (Na_2_SO_4_) exert differential effects on growth, chlorophyll content, proline accumulation, and oxidative stress. These responses vary depending on species, genotype, salt type, ionic composition, concentration, and exposure duration (Liu et al., 2025; Wang et al., 2023). However, comparative studies evaluating the ionic specificity of different salt types in *L. perenne* are extremely limited, and most existing research focuses solely on NaCl. This creates a significant knowledge gap regarding how distinct anions and cations shape physiological responses and antioxidant gene expression under salinity. Despite these variations among salt types, salinity ultimately converges on a common mechanism: the excessive accumulation of reactive oxygen species (*ROS*) in root and leaf tissues, leading to oxidative damage and metabolic imbalances. Plants counteract these effects by activating complex defense systems that include both enzymatic and non-enzymatic antioxidant components, such as the ascorbate–glutathione (AsA–GSH) cycle and the antioxidant enzymes superoxide dismutase (*SOD*), catalase (*CAT*), and peroxidase (*POD*) (Kesawat et al., 2023; Abubakar et al., 2023). For this reason, the genes APX, GR, PCS, and HMA were selected because they play central roles in oxidative stress detoxification, redox regulation, and ion homeostasis under salinity.

Calcium ions (Ca²^+^) play a pivotal role as secondary messengers in maintaining ionic homeostasis particularly the Na^+^/K^+^ ratio and in regulating antioxidant gene responses under saline conditions (Kurusu et al., 2015; Seifikalhor et al., 2019).

Within the AsA–GSH cycle, ascorbate peroxidase (*APX*) detoxifies H_2_O_2_ using ascorbate as an electron donor, while glutathione reductase (*GR*) regenerates reduced glutathione (GSH) from its oxidized form, thereby maintaining the continuity of the cycle (Seckin et al., 2010). For this reason, APX and GR were included as key antioxidant markers, while PCS and HMA were selected due to their roles in detoxification, redox regulation, and ion homeostasis under salinity.

Studies conducted on rice (*Oryza sativa*) roots have demonstrated that salt treatment enhances ascorbate peroxidase (*APX*) and glutathione reductase (*GR*) enzyme activities, as well as the expression of *OsAPX* and *OsGR* genes, suggesting that salinity triggers reactive oxygen species (ROS)-mediated signaling pathways (Tsai et al., 2005). Similarly, in perennial ryegrass (*Lolium perenne* L.), exposure to 250 mM sodium chloride (NaCl) significantly alters antioxidant enzyme activities, isoform composition, and gene expression profiles, including those of *APX* and *GR* (Hu et al., 2012). It has been reported that the hydrogen peroxide (H_2_O_2_)–calcium ion (Ca²^+^) signaling axis plays a pivotal role in salt tolerance in *L. perenne*, where enhanced antioxidant enzyme activity and maintenance of the potassium/sodium (K^+^/Na^+^) ratio contribute to physiological stability (Miao et al., 2022). Exogenous applications of Ca²^+^ and magnesium ion (Mg²^+^) have been shown to strengthen plasma membrane and cell wall stability, maintain ionic balance, and enhance the efficiency of antioxidant defense systems, thereby improving salt tolerance (Bahmani et al., 2015; Sharavdorj et al., 2022; Mumtaz et al., 2025). Phytochelatin synthase (*PCS*), initially identified in heavy metal detoxification, has also been implicated in oxidative and salt stress responses (Clemens et al., 1999; Singh et al., 2016; Maryam et al., 2025). In addition, the expression of heavy metal ATPases (*HMAs*), a group of membrane transport proteins, can be indirectly modulated by various salt treatments such as NaCl, calcium chloride (CaCl_2_), and magnesium chloride (MgCl_2_) through mechanisms involving Ca²^+^ signaling and membrane transporter stabilization (Seifikalhor et al., 2019; Ahmad et al., 2025).

Most previous studies on perennial ryegrass (*Lolium perenne* L.) have primarily focused on sodium chloride (NaCl)-induced salt stress, examining either physiological traits or a limited number of antioxidant-related genes. In the present study, the expression of salt stress–responsive genes ascorbate peroxidase (*APX*), glutathione reductase (*GR*), heavy metal ATPase (*HMA*), and phytochelatin synthase (*PCS*) was evaluated under treatments with calcium chloride (CaCl_2_), magnesium chloride (MgCl_2_), magnesium sulfate (MgSO_4_), and sodium sulfate (Na_2_SO_4_). The aim was to investigate how different salt types influence gene expression and physiological responses, thereby addressing a critical gap in the current understanding of salt tolerance mechanisms in *L. perenne*.

Seedlings of *L. perenne* were exposed to five salt concentrations (30, 40, 60, 70, and 90 mM) of CaCl_2_, MgCl_2_, MgSO_4_, and Na_2_SO_4_. Gene expression levels of *APX*, *GR*, *HMA*, and *PCS* were quantified using quantitative real-time polymerase chain reaction (qRT-PCR). While these salts were primarily used to induce salinity stress, their constituent ions (e.g., calcium (Ca²^+^) and magnesium (Mg²^+^)) also play essential roles in plant nutrition and stress signaling.

The overall objective of this study was to elucidate the molecular responses of perennial ryegrass to different chemical sources of salinity and to contribute to a better understanding of salt tolerance mechanisms through gene expression profiling. Additionally, agronomic traits such as seedling height, fresh and dry biomass, and water content were measured to complement the molecular findings with morphological indicators.

In this context, the present study addresses the following research question: How do different salt types with distinct ionic compositions modulate antioxidant gene expression (*APX, GR, HMA, PCS*) and agronomic traits in perennial ryegrass seedlings? We hypothesized that salts differing in their ionic composition (Ca²^+^-, Mg²^+^-, and Na^+^-based sources) would elicit distinct gene expression patterns and physiological responses due to differences in their ionic and signaling properties.

## 2 Materials and methods

### 2.1 Plant material and experimental design in *Lolium perenne*

This experiment was conducted in March 2024 under greenhouse conditions in Pazar district, Rize province, Türkiye. The plant material consisted of commercially sourced seeds of *Lolium perenne* L. (Brand: Grass Seed^®^, Green World, 100% *Lolium perenne* L., Türkiye). The product contained exclusively *L. perenne* without any admixture of other grass species. Seeds were used directly without any pre-selection or purification processes. The commercial cultivar was used without any prior selection, and it represents a commonly cultivated form of *Lolium perenne*.

**Figure 1:**
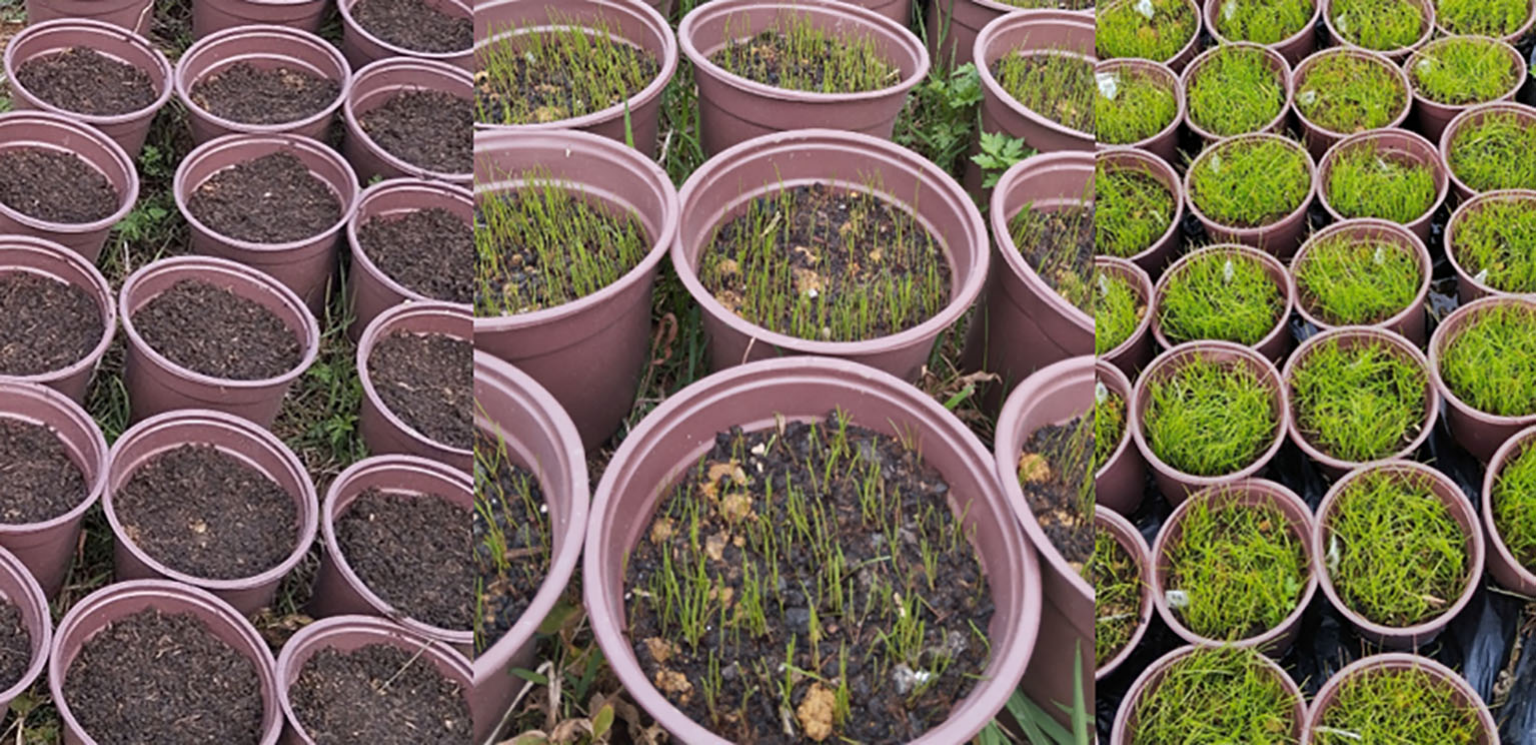
Early germination and tillering stages of *Lolium perenne* plants grown in pots.

The trial was established in plastic pots (16 × 13 cm) filled with sterilized commercial peat (pH 6.0) supplemented with 1.0 g/L fertilizer. A factorial experiment was arranged in a randomized block design with three replications. Seeds were sown by broadcasting and covered with a thin layer of peat. For the first 30 days, all pots were irrigated uniformly with distilled water under identical environmental conditions (temperature, light, humidity, and irrigation), and no salt treatments were applied (**Figure 1**).

From day 30 onward, salinity treatments were imposed using CaCl_2_, MgCl_2_, MgSO_4_, and Na_2_SO_4_ at concentrations of 0 (control), 30, 40, 60, 70, and 90 mM, resulting in a total of 20 treatment groups, each represented by three biological replicates (Bonnin et al., 2023). Each pot received 500 mL of the respective salt solution at a single application, whereas the control group received only distilled water.

Agronomic measurements were taken using a precision balance and a digital caliper, while dry weights were determined after drying at 78°C for 48 h. Plants were harvested 30 days after the initiation of salt treatments, and agronomic traits were recorded separately for each replication. For genetic analyses, three biological replicates were pooled to form composite samples, and molecular studies were conducted on these composites.

### 2.2 RNA isolation and cDNA synthesis

Following the completion of the 20 treatment groups described in Section 2.1, three biological replicates from each group were pooled to obtain one composite sample for molecular analysis. Total RNA was extracted from 300 mg of *Lolium perenne* leaf tissue using the Ecotech Plant Total RNA Kit (Ecotech, Türkiye). Leaf samples were ground in liquid nitrogen, homogenized with lysis buffer, and subjected to chloroform-based phase separation. The aqueous phase was collected, RNA was precipitated with isopropanol, passed through a membrane column, washed with the kit buffers, and eluted in 50 µL of RNase-free elution buffer. RNA samples were stored at –20°C until use. cDNA synthesis was performed from 100 ng of total RNA using the Ecotech 5× First Strand cDNA Synthesis Kit, strictly following the manufacturer’s protocol (42°C for 100 min and 85°C for 5 min), which specifies the extended incubation period. The synthesized cDNA was stored at –20°C. All reactions were conducted using a conventional PCR thermal cycler (Thermo Scientific, USA).

### 2.3 qRT-PCR analysis

cDNA synthesis was performed using 100 ng of total RNA with the Ecotech 5× First Strand cDNA Synthesis Kit (Ecotech, Türkiye). The reaction consisted of incubation steps at 42°C for 100 min and 85°C for 5 min. The synthesized cDNA samples were stored at –20°C for subsequent analyses. All reactions were carried out in a conventional PCR thermal cycler (Thermo Scientific, USA).

Before cDNA synthesis, RNA samples were spectrophotometrically quantified and adjusted to a final concentration of 100 ng/µL. All synthesized cDNA samples were subsequently diluted to the same concentration and used for qPCR analyses.

Amplification was performed using a Roche LightCycler 96 system (Roche, Germany). The cycling protocol included an initial denaturation at 95°C for 15 min, followed by 40 cycles of 95°C for 15 s (denaturation), primer-specific annealing temperatures for 30 s (as listed in **Table 1**), and 72°C for 30 s (extension). A final denaturation step at 95°C for 10 s was followed by a melting curve analysis.

**Table 1:** Forward and reverse primer sequences used for the amplification of *Lolium perenne* genes.

Genes	Forward sequences (5’-3’)	Reverse sequences (5’-3’)	TM (°C)
*β-actin*	CTTCGCGGGCGACGAT	CACATAGGAATCCTTCTGACCCAT	59
*HMA*	TTCCCCACAAGAATCGCTCC	CACTCGAACCTTCCACGTCA	59
*PCS*	CACAGACATGGTCAGGGAT	AAGCATAGTTGGGAGGGA	56
*GR*	TGTGCTGTTTTCTGCATTCC	AGTCTCAGCATCAACCACCA	56
*APX*	CCTGAAAGGTCTGGGTTTGA	TCCTTGGCATAAAGGTCCAC	56

Gene sequences were retrieved from the NCBI database, and primers required for RT-PCR were designed using the NCBI Primer-BLAST tool (https://www.ncbi.nlm.nih.gov/tools/primer-blast).

The mRNA expression levels of salt stress–related genes (*HMA*, *PCS*, *GR*, and *APX*) were analyzed by qRT-PCR. β-Actin was used as the internal housekeeping control gene. The forward and reverse primer sequences for *Lolium perenne* genes are listed in **Tables 1** and **2**. For each primer pair, the corresponding annealing temperature (Tm) is also indicated in the primer table to ensure clarity and reproducibility of the qRT-PCR analyses.

**Table 2:** RNA quantity (ng/µL), 260/280 ratio, and Cycle Threshold (Ct) values obtained from qRT-PCR analyses of salinity-stressed perennial ryegrass (*Lolium perenne*) samples (n = 20).

Samples (1-20)	RNA amounts (ng/μl)	260/280 ratio (nm)	*β-actin* (Ct)	*APX* (Ct)	*GR* (Ct)	*HMA* (Ct)	*PCS* (Ct)
Control	217.36	1.97	35.35	27.74	34.99	38.24	37.71
CaCl_2_ (30mM)	368.18	2.01	33.05	25.36	33.73	36.41	35.90
CaCl_2_ (40mM)	315.04	1.95	34.45	29.68	36.51	38.78	41.94
CaCl_2_ (60mM)	473.69	2.00	33.22	29.70	34.71	40.44	38.39
CaCl_2_ (70mM)	234.21	1.92	32.92	27.09	34.73	36.00	38.44
CaCl_2_ (90mM)	313.30	1.93	31.44	26.21	33.31	35.63	36.16
MgCl_2_ (30mM)	454.90	2.06	33.69	27.17	34.31	38.10	37.97
MgCl_2_ (40mM)	487.34	2.01	31.95	28.59	34.70	39.65	37.38
MgCl_2_ (60mM)	357.40	2.00	33.45	27.46	33.41	37.48	38.37
MgCl_2_ (70mM)	369.35	1.97	34.72	27.22	35.36	37.95	38.68
MgCl_2_ (90mM)	243.12	1.97	34.86	25.66	35.6	39.00	36.92
MgSO_4_ (30mM)	681.87	2.08	33.29	26.33	34.17	40.77	38.64
MgSO_4_ (40mM)	519.76	2.06	32.56	26.98	34.24	36.77	39.07
MgSO_4_ (60mM)	486.74	1.88	33.98	29.96	33.38	41.43	40.67
MgSO_4_ (70mM)	470.10	2.02	32.64	25.94	34.29	37.42	37.40
MgSO_4_ (90mM)	370.95	2.09	30.93	27.39	33.96	34.80	34.65
Na_2_SO_4_ (30mM)	620.62	2.04	32.53	28.92	37.82	38.94	38.21
Na_2_SO_4_ (40mM)	490.98	2.02	32.66	27.63	33.99	38.49	37.25
Na_2_SO_4_ (60mM)	602.68	2.09	33.21	27.02	33.73	37.03	36.09
Na_2_SO_4_ (70mM)	612.44	2.07	32.59	27.98	34.29	38.94	37.24

In qPCR analyses, the cycle threshold (Ct) value was defined as the number of cycles required for the fluorescent signal to exceed the minimum detection threshold (**Table 2**). Relative gene expression levels were calculated using the 2^−ΔΔCT^ method (Livak and Schmittgen, 2001). The calculations were performed according to the following steps. For comparative evaluation of salt stress–related gene expressions (*APX, GR, HMA*, and *PCS*) in *Lolium perenne* samples (1–20) relative to the control group, one-way ANOVA was conducted using Prism 9 software (GraphPad, USA). To determine significant differences between the control and multiple gene groups, Dunnett’s *post hoc* test was applied.

### 2.3 Agronomic measurements

Seedling length was measured using a digital caliper, and the averages were calculated (Muratbek Kyzy et al., 2023; Yıldırım and Yılmaz, 2023; Sohail et al., 2025). Seedling fresh and dry weights were measured using a precision scale, and their averages were recorded. The plant water content and dry matter content were calculated using the following formulas:


$$Plant\;Water\;Content(\%):\left(\frac{Fresh\;Weight-Dry\;Weight}{Fresh\;Weight}\right)\times 100$$

$$Dry\;Matter\;Content(\%):\left(\frac{Dry\;Weight}{Fresh\;Weight}\right)\times 100$$

### 2.4 Statistical analysis

Statistical analyses were conducted on agronomic measurements to determine the significance of the experimental data and the sources of variation. The analysis was performed using JMP software. The Tukey test, a multiple comparison method, was used to evaluate whether the differences between the groups were statistically significant.

## 3 Results and discussion

### 3.1 qRT-PCR analysis of salinity stress activities in *Lolium perenne*

Gene expression activities related to salt stress (*APX, GR, HMA, PCS*) in *Lolium perenne* samples were analyzed using the qRT-PCR method. β-actin was employed as the reference (housekeeping) gene, while untreated plant tissues were used as negative controls.

**Figure 2:**
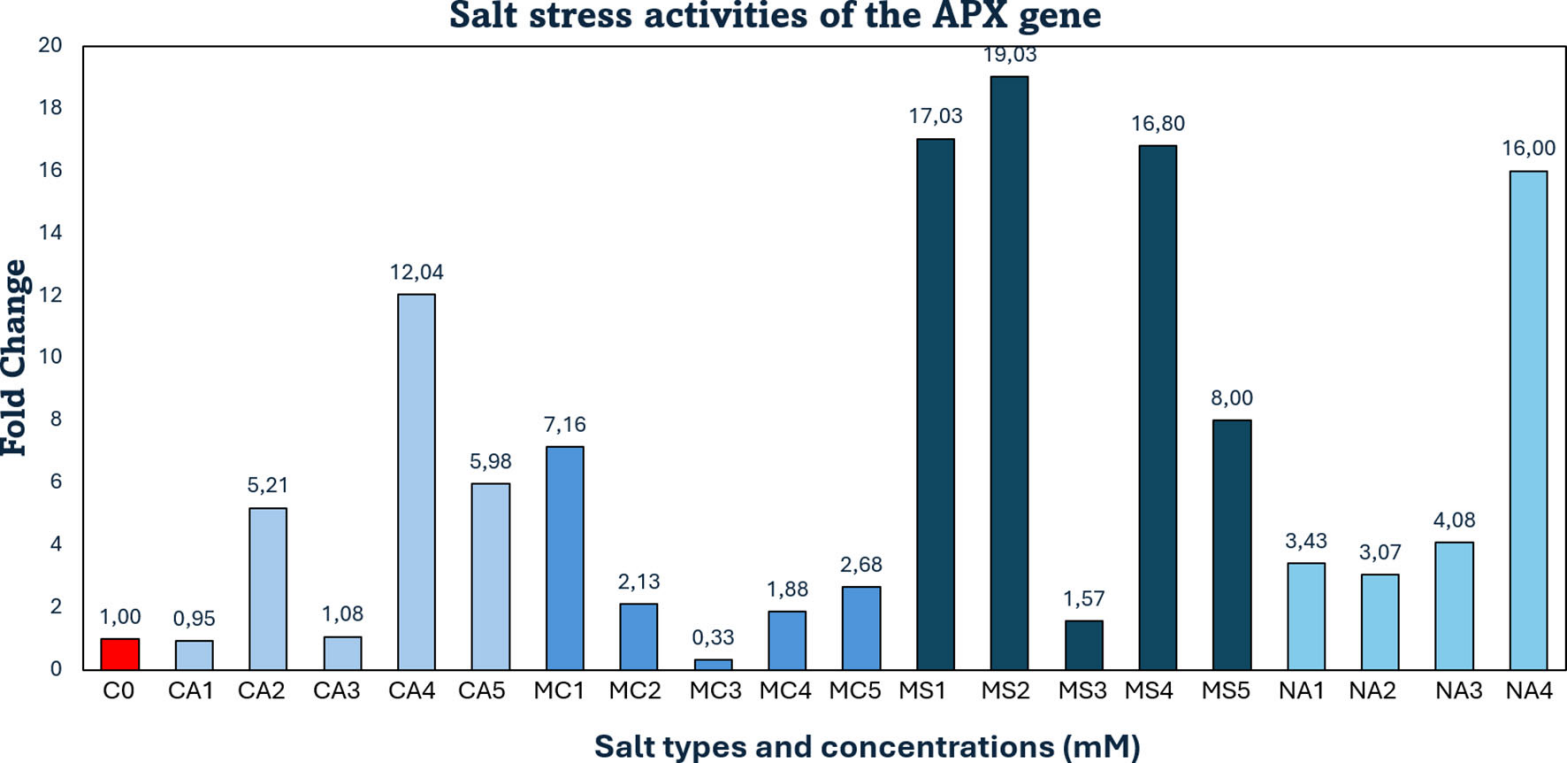
qRT-PCR mRNA expression levels of the *APX* salt-stress–responsive gene in *Lolium perenne*, C0 = Control (non-stressed); CA1–CA5 = CaCl_2_ (30, 40, 60, 70, 90 mM); MC1–MC5 = MgCl_2_ (30, 40, 60, 70, 90 mM); MS1–MS5 = MgSO_4_ (30, 40, 60, 70, 90 mM); NA1–NA4 = Na_2_SO_4_ (30, 40, 60, 70 mM); *APX* = Ascorbate Peroxidase.

Under salt stress conditions, *APX* gene expression decreased by 5% (0.05-fold) following treatment with 30 mM CaCl_2_ compared to the control. At 60 mM CaCl_2_, the expression was markedly suppressed, showing no upregulation. However, significant inductions were observed at higher concentrations: 5-fold at 40 mM, 12-fold at 70 mM, and 6-fold at 90 mM CaCl_2_. In MgCl_2_ treatments, a slight downregulation (3%) occurred at 30 mM, whereas expression increased by 15% and 24% at 40 and 70 mM, respectively. Conversely, 60 mM MgCl_2_ caused a pronounced reduction in expression (67%; 0.7-fold). Although 40, 70, and 90 mM MgCl_2_ also resulted in mild upregulation (2.1-, 1.8-, and 1.6-fold, respectively), a sharp induction (7.1-fold) was detected at 30 mM. With 60 mM MgSO_4_, *APX* expression showed a modest increase (1.5-fold), indicating tolerance under stress. In contrast, substantial upregulation was recorded at 30, 40, 70, and 90 mM MgSO_4_ (17-, 19-, 16-, and 8-fold, respectively). Likewise, in Na_2_SO_4_ treatments, *APX* expression peaked at 70 mM with a 16-fold increase. Notably, 30-, 40-, and 60-mM concentrations also induced expression by approximately 3- to 4-fold (**Figure 2**).

These findings align closely with previous reports stating that *CAT, POD, APX, GPX* and *GR* genes are upregulated in perennial ryegrass under salt stress, forming a crucial part of the antioxidative defense mechanism against ROS accumulation (Miao et al., 2022; Yang et al., 2019). Consistently, Wu et al. (2018) reported significantly elevated *APX* activity in transgenic lines of perennial ryegrass exposed to 400 mM NaCl, as compared to wild-type plants. This enhancement facilitated more efficient ROS scavenging within chloroplasts, thereby contributing to the maintenance of physiological stability under salt stress.

Compared with the control, *GR* gene expression decreased by approximately 15% (0.15-fold) under salt stress at 70 mM CaCl_2_. In contrast, treatments with 30, 40, 60, and 90 mM CaCl_2_ resulted in 2- to 4-fold increases in *GR* transcript levels. Similarly, exposure to 30–90 mM MgCl_2_ led to 2- to 5-fold upregulation across the tested concentrations.

**Figure 3:**
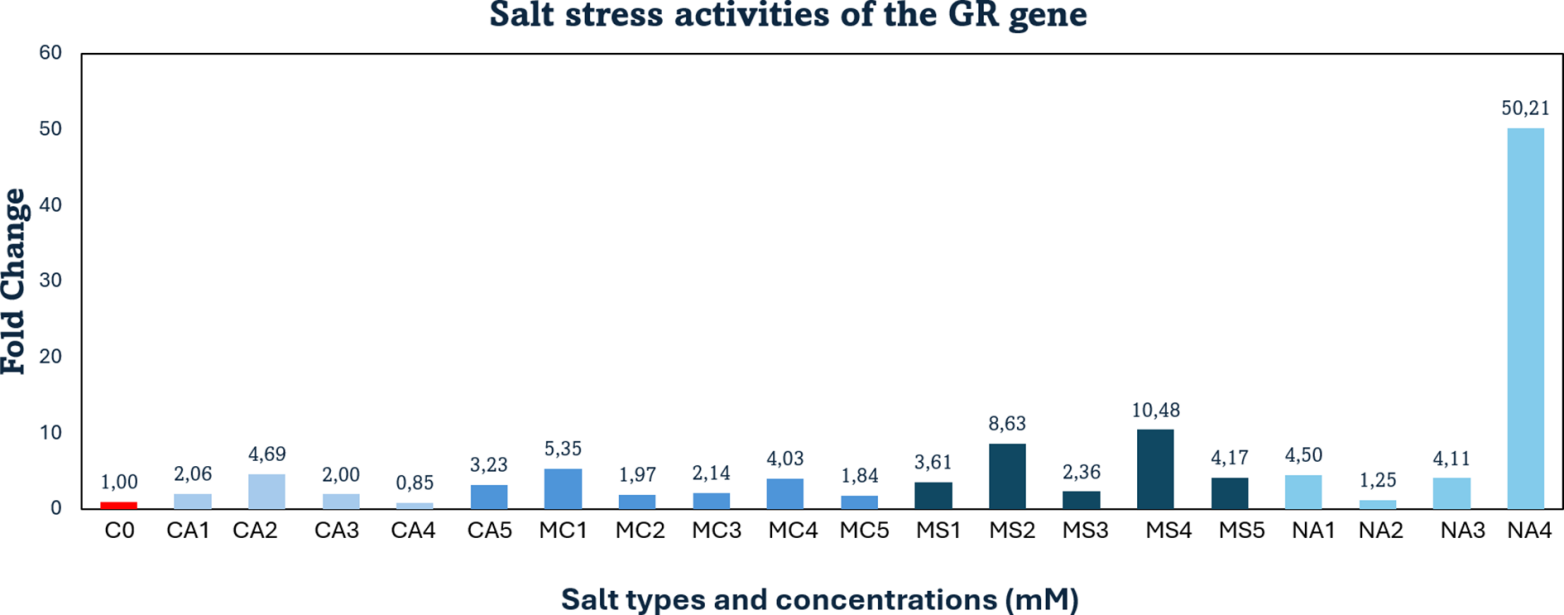
qRT-PCR mRNA gene expression levels of the *GR* salt stress gene in *Lolium perenne* samples, C0, Control (non-stressed); CA1–CA5 = CaCl_2_ (30, 40, 60, 70, 90 mM); MC1–MC5 = MgCl_2_ (30, 40, 60, 70, 90 mM); MS1–MS5 = MgSO_4_ (30, 40, 60, 70, 90 mM); NA1–NA4 = Na_2_SO_4_ (30, 40, 60, 70 mM); *GR* = Glutathione Reductase.

For MgSO_4_ treatments, the highest expression levels were recorded at 40 and 70 mM, showing 8- and 10-fold increases, respectively. Moderate increases (2–4-fold) were also detected at 30, 60, and 90 mM MgSO_4_. Under 70 mM Na_2_SO_4_, the *GR* gene was strongly upregulated by 50-fold, whereas a mild enhancement (1.2-fold) was observed at 40 mM Na_2_SO_4_, indicating stress tolerance at this level. At 30 and 60 mM Na_2_SO_4_, *GR* expression rose approximately 4-fold (**Figure 3**).

Comparable results were reported by Meena et al. (2023), who demonstrated that *GR* and *GPX* expression levels significantly increased with rising salinity intensity in inoculated plants; at 15 dS m^-^¹ salinity, *GR* and *GPX* were upregulated 8- and 9-fold, respectively. These findings support the consistency of the *GR* gene response under salt stress with the current study.

Similarly, in tomato varieties (*Solanum lycopersicum*), Horváth et al. (2023) investigated genotype-specific expression of *GR* and *GST* genes and reported that salt tolerance levels were closely associated with such genetic differences. The salt-sensitive cultivar Mobil exhibited low *GR* and *GST* activities and a weak redox balance, while Moneymaker and Elán F1 maintained growth through enhanced *GR* activity and antioxidant defense responses. The study also highlighted the critical role of *GR* and certain transcription factors (*WRKY3, WRKY72, DREB1/2*) in regulating redox homeostasis under salinity stress. The observed increase in *GR* gene expression in the present study is therefore consistent with previous findings, suggesting that elevated *GR* activity contributes to maintaining redox equilibrium and enhancing salt tolerance.

Compared to the control, *HMA* gene expression under salt stress was upregulated 20-fold in response to 70 mM CaCl_2_. In contrast, treatments with 30, 40, and 60 mM CaCl_2_ resulted in only slight increases (1.3-, 2.4-, and 1.2-fold, respectively), and stress appeared to be tolerated at these levels.

Under 30–90 mM MgCl_2_ treatments, *HMA* expression also increased modestly, ranging between 1.9- and 3.7-fold, indicating a tolerant response. In the MgSO_4_ treatments, only the 70 mM dose resulted in a mild increase (1.9-fold), with stress tolerance observed. However, substantial upregulation was observed at 30, 40, 60, and 90 mM MgSO_4_, with 40-, 28-, 24-, and 11-fold increases, respectively.

**Figure 4:**
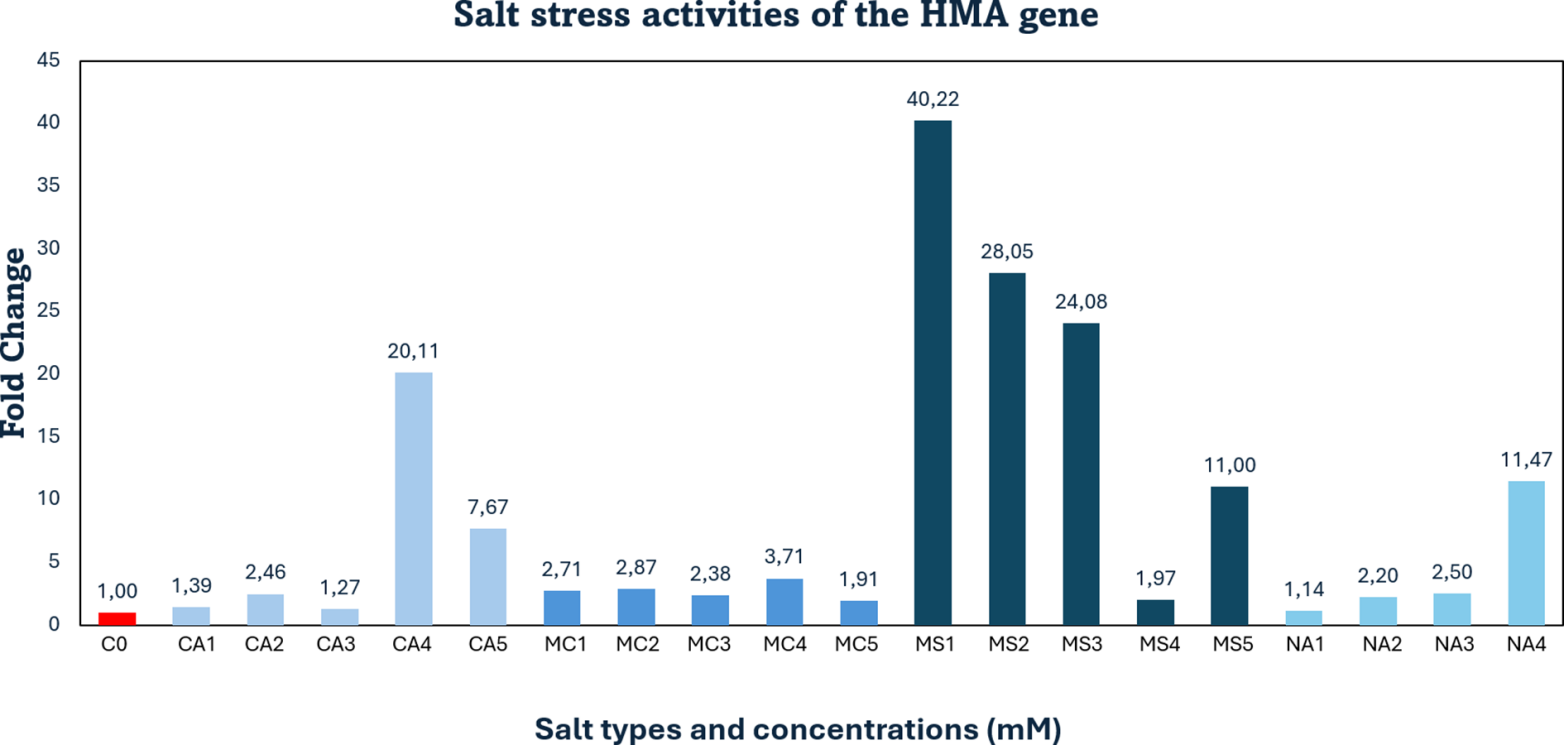
qRT-PCR mRNA gene expression levels of the *HMA* salt stress gene in *Lolium perenne* samples, C0, Control (non-stressed); CA1–CA5 = CaCl_2_ (30, 40, 60, 70, 90 mM); MC1–MC5 = MgCl_2_ (30, 40, 60, 70, 90 mM); MS1–MS5 = MgSO_4_ (30, 40, 60, 70, 90 mM); NA1–NA4 = Na_2_SO_4_ (30, 40, 60, 70 mM); *HMA* = Heavy Metal ATPase.

As for Na_2_SO_4_, *HMA* expression increased 11-fold at the 70 mM dose. At 30, 40, and 60 mM Na_2_SO_4_, gene expression was upregulated to a lesser extent (1.1-, 2.2-, and 2.5-fold, respectively), and tolerance was evident (**Figure 4**).

Although *HMA* genes are primarily studied for their roles in heavy metal transport and homeostasis, several reports indicate their partial activation under salt-induced stress conditions. For example, in Mesembryanthemum crystallinum (ice plant), a study by Nosek et al. (2020) demonstrated that NaCl-induced salt stress significantly upregulated the *hma4* (Heavy Metal ATPase 4) gene, particularly in root tissues. This upregulation was confirmed by comparing salt-treated plants with controls not exposed to Cd or NaCl, and NaCl treatment was shown to directly induce *hma4* expression. This observation supports the current findings, where salt-induced *HMA* gene expression was also markedly elevated.

Compared to the control, *PCS* gene expression under salt stress was strongly upregulated by 20-fold following treatment with 70 mM calcium chloride (CaCl_2_). A slight increase (1.4-fold) was observed at 30 mM CaCl_2_, indicating that stress was tolerated at this concentration. Similarly, treatments with 40, 60, and 90 mM CaCl_2_ led to increases of 5.1-, 3-, and 4.6-fold, respectively.

Under 60 mM magnesium chloride (MgCl_2_), *PCS* expression decreased by approximately 18% (0.19-fold). In contrast, a mild upregulation (1.4-fold) was observed at 90 mM MgCl_2_, indicating tolerance. The highest expression level was recorded at 30 mM MgCl_2_, with a dramatic 35-fold increase. Additionally, 40 and 70 mM MgCl_2_ treatments resulted in 3.7- and 5.2-fold increases, respectively.

In magnesium sulfate (MgSO_4_) treatments, a slight increase (2.5-fold) was detected at 70 mM, suggesting stress tolerance. Similarly, gene expression was upregulated by 7-, 8.4-, 7.9-, and 4.9-fold under 30, 40, 60, and 90 mM MgSO_4_, respectively.

**Figure 5:**
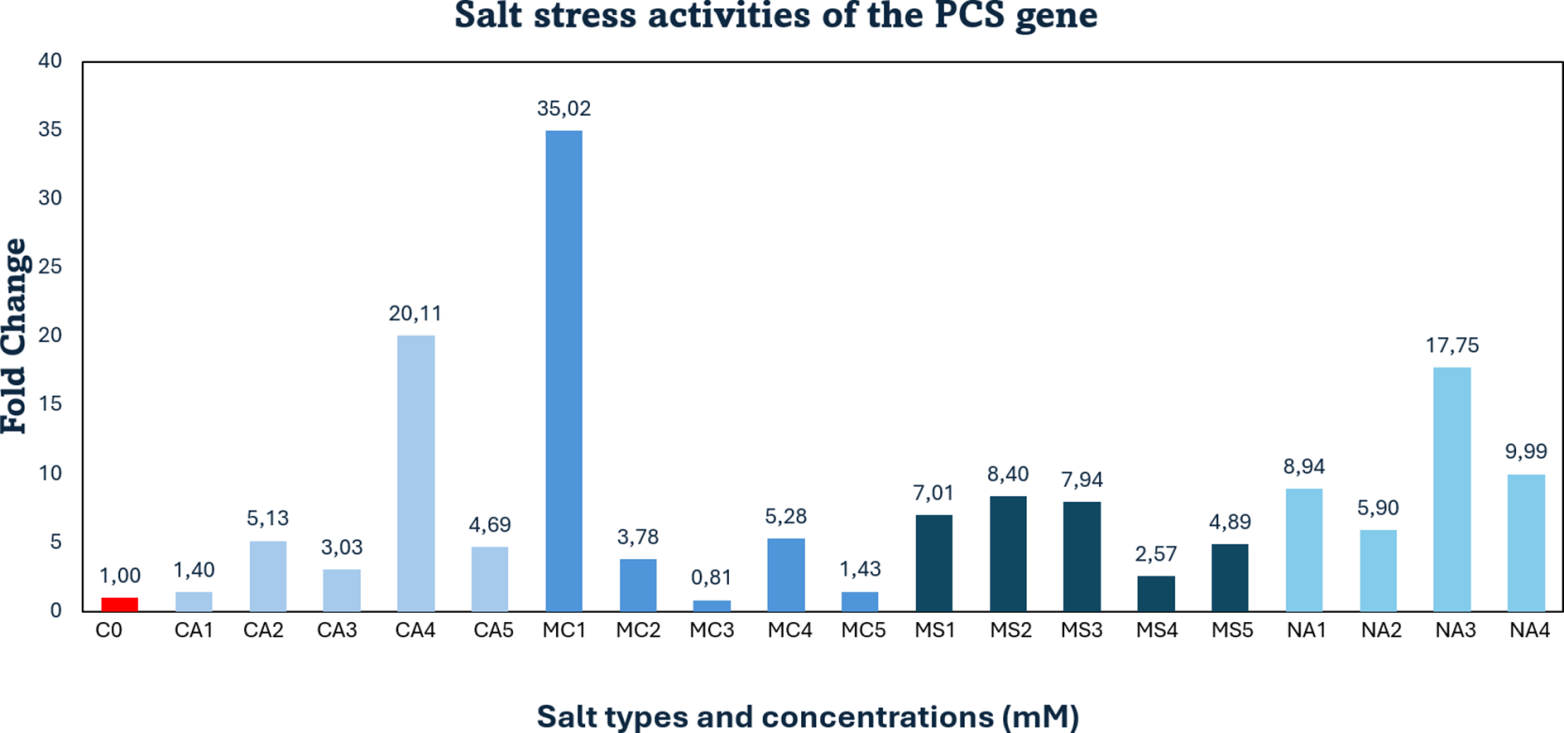
qRT-PCR mRNA gene expression levels of the *PCS* salt stress gene in *Lolium perenne* samples, C0, Control (non-stressed); CA1–CA5 = CaCl_2_ (30, 40, 60, 70, 90 mM); MC1–MC5 = MgCl_2_ (30, 40, 60, 70, 90 mM); MS1–MS5 = MgSO_4_ (30, 40, 60, 70, 90 mM); NA1–NA4 = Na_2_SO_4_ (30, 40, 60, 70 mM); *PCS* = Phytochelatin Synthase.

Under 60 mM sodium sulfate (Na_2_SO_4_), *PCS* expression showed a strong upregulation of 17-fold. Furthermore, 8.9-, 5.9-, and 9.9-fold increases were recorded at 30, 40, and 70 mM Na_2_SO_4_, respectively (**Figure 5**).

In line with our findings, Kim et al. (2019) demonstrated that the *AtPCS2* gene in *Arabidopsis thaliana* was significantly upregulated under 100 and 200 mM NaCl treatments, and transgenic plants exhibited enhanced tolerance to salt stress. Similarly, Su et al. (2020) reported that in Ipomoea pes-caprae, *PCS* gene expression was primarily associated with metal stress, but also responsive to various abiotic stress factors, indicating broader functional relevance beyond metal detoxification.

**Figure 6:**
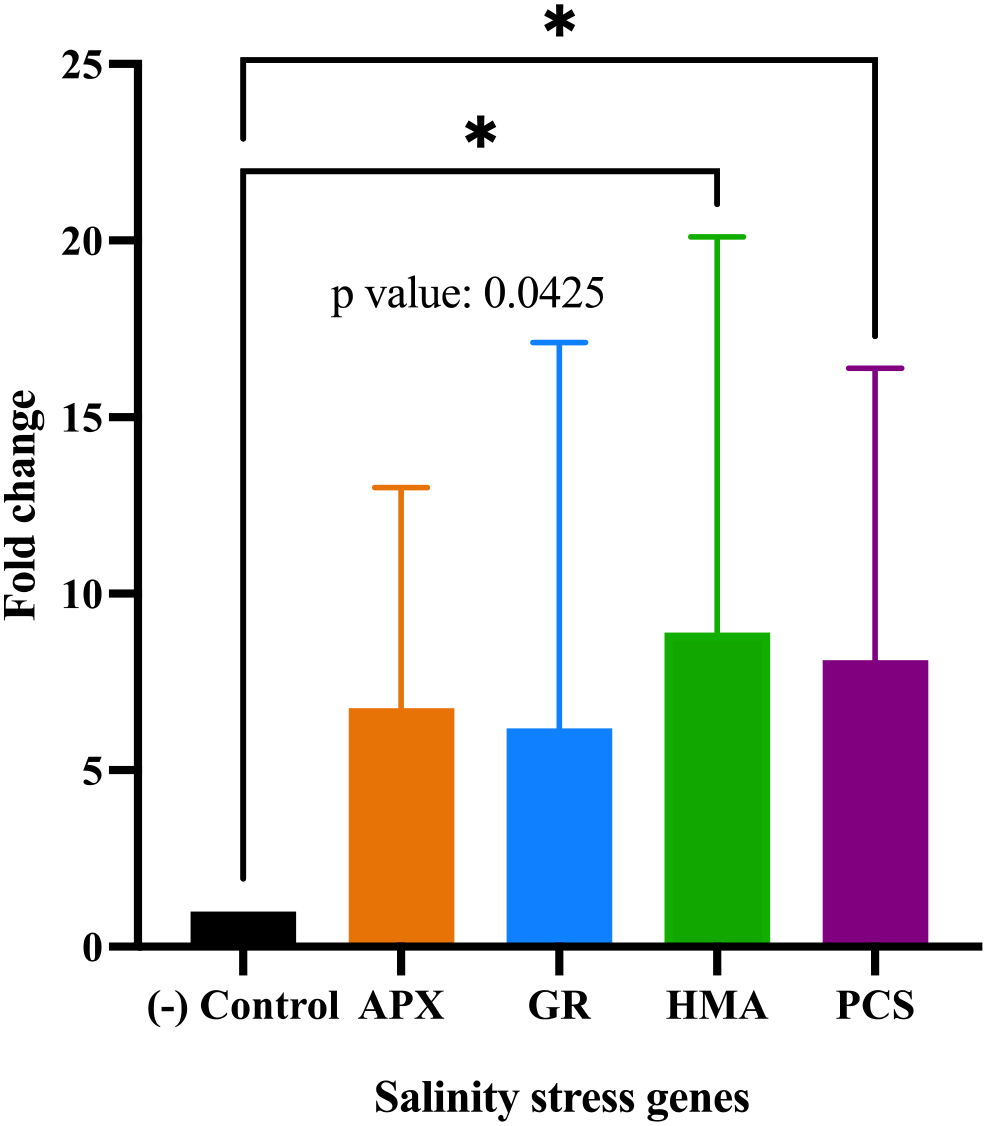
One-way ANOVA analysis of salt stress gene expressions (*APX, GR, HMA, PCS*) of *Lolium perenne* samples (1–19), where sample 1 represents the non-stressed control (n = 20), compared to salt-stressed groups by qRT-PCR (p< 0.05). Bars represent mean fold change ± SE. Asterisks indicate significant differences compared to the control (*p< 0.05, exact p = 0.0425*).

In this study, downregulation of *APX*, *GR*, and *PCS* gene expression was observed at certain high salt concentrations, indicating that the plants were able to tolerate stress under these conditions. Specifically, *APX* expression decreased to 0.05-fold (5%) with 30 mM calcium chloride (CaCl_2_) and to 0.7-fold (67%) with 60 mM magnesium chloride (MgCl_2_). The *GR* gene was downregulated to 0.15-fold (15%) under 70 mM CaCl_2_, while the *PCS* gene showed a 0.19-fold (18%) decrease at 60 mM MgCl_2_. Overall, strong upregulation responses were detected in *APX, GR, HMA* and *PCS* genes under various salt treatments. Specifically: *APX* was upregulated 12-fold at 70 mM CaCl_2_, 7.1-fold at 30 mM MgCl_2_, 17-, 19-, and 16-fold at 30, 40, and 70 mM MgSO_4_, respectively, and 16-fold at 70 mM Na_2_SO_4_. *GR* expression increased 10-fold at 70 mM MgSO_4_ and 50-fold at 70 mM Na_2_SO_4_. *HMA* showed a 20-fold increase at 70 mM CaCl_2_ and was upregulated 40-, 28-, 24-, and 11-fold at 30, 40, 60, and 90 mM MgSO_4_, respectively, as well as 11-fold at 70 mM Na_2_SO_4_. *PCS* expression rose 20-fold at 70 mM CaCl_2_, 35-fold at 30 mM MgCl_2_, and 17-fold at 60 mM Na_2_SO_4_. As a result, a one-way ANOVA (Dunnett’s multiple comparison test) revealed that the expression levels of *HMA* and *PCS* genes under salt stress (treatments 1–19) in *Lolium perenne* differed significantly from the control group (p = 0.0425) (**Figure 6**).

### 3.2 Agronomic measurements

**Figure 7:**
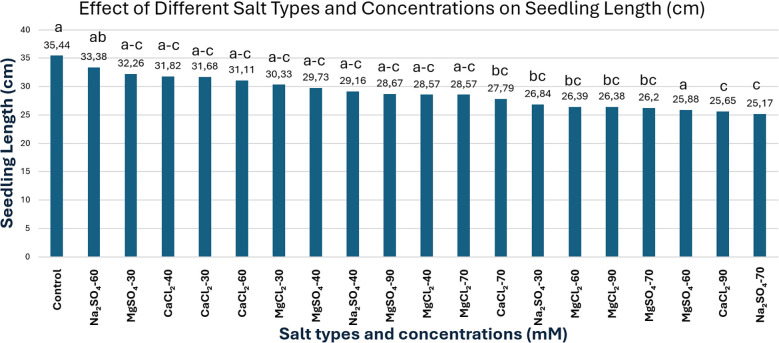
Effect of different salt types and concentrations on seedling length (cm). Means followed by the same letters are not significantly different according to Tukey’s multiple comparison test (p ≤ 0.05). Overall, treatment effects were statistically highly significant (p ≤ 0.001), with a coefficient of variation (CV%) of 8.41.

The control group exhibited the highest seedling height with 35.44 cm, which was found to be statistically different from all salt treatment groups. These results clearly indicate the detrimental impact of salt compounds on the growth performance of *Lolium perenne*, and this pattern becomes more pronounced as salt concentration increases. Although the treatments of 60 mM sodium sulfate (33.38 cm), 30 mM magnesium sulfate (32.26 cm), and 40 mM calcium chloride (31.82 cm) resulted in noticeable reductions in seedling height, no statistically significant differences were observed among these treatments (**Figure 7**). Moreover, the severity of growth inhibition varied across treatments. In contrast, the 90 mM calcium chloride (25.65 cm) and 70 mM sodium sulfate (25.17 cm) treatments caused the most severe reductions in seedling height, demonstrating that high salt concentrations more strongly inhibit plant growth. The coefficient of variation (CV = 8.41%) indicated a moderate level of variability among treatments.

These findings are consistent with previous studies investigating the effects of various salt compounds on plant growth. Numerous reports have documented that salinity stress limits growth in *Lolium perenne* and other salt-sensitive species (Roohollahi and Kafi, 2010; Nizam, 2011; Kusvuran et al., 2015; Ren et al., 2025; Yılmaz and Kısakürek, 2018; Tatar et al., 2018; Alagöz and Türk, 2020; Kıldış, 2021). In particular, Xu et al. (2020) extensively examined the physiological responses of *L. perenne* under saline conditions, reporting that high salinity levels caused pronounced seedling wilting and increased mortality rates in a concentration-dependent manner, thereby severely impairing physiological functioning and limiting plant survival capacity.

Among the treatments, 40 mM sodium sulfate yielded the highest fresh weight (0.18 g), which was statistically higher than that of all other treatment groups. This result suggests that moderate concentrations of sodium sulfate exert less damaging effects on water retention capacity and tissue integrity in *L. perenne* seedlings. In contrast, treatments with 60 mM sodium sulfate (0.10 g), 30 mM magnesium sulfate (0.11 g), and 60 mM calcium chloride (0.09 g) produced fresh weights comparable to the control group (0.09 g), with no statistically significant differences observed. These findings indicate that, particularly at low to moderate salt concentrations, some treatments did not markedly impair fresh biomass accumulation (**Table 3**).

**Table 3:** Means and significance levels of seedling fresh weight and seedling dry weight.

Treatments	S.F.W. (g)	Treatments		S.D.W. (g)
Na_2_SO_4_-40	0.18	MgSO_4_-30	a	0.04
MgSO_4_-30	0.11	Control	a	0.04
Na_2_SO_4_-60	0.10	MgSO_4_-60	a	0.04
CaCl_2_-60	0.09	Na_2_SO_4_-40	ab	0.03
Control	0.09	Na_2_SO_4_-70	ab	0.03
CaCl_2_-40	0.08	MgSO_4_-40	ab	0.03
CaCl_2_-70	0.08	Na_2_SO_4_-60	ab	0.03
MgSO_4_-70	0.08	CaCl_2_-40	ab	0.03
Na_2_SO_4_-70	0.07	CaCl_2_-70	ab	0.03
MgCl_2_-30	0.07	CaCl_2_-30	b	0.02
MgCl_2_-40	0.07	MgCl_2_-40	b	0.02
MgSO_4_-40	0.07	MgCl_2_-60	b	0.02
CaCl_2_-30	0.06	MgCl_2_-70	b	0.02
MgCl_2_-70	0.06	MgSO_4_-90	b	0.02
MgSO_4_-60	0.06	CaCl_2_-60	b	0.02
MgSO_4_-90	0.06	MgCl_2_-90	b	0.02
Na_2_SO_4_-30	0.06	Na_2_SO_4_-30	b	0.02
MgCl_2_-60	0.06	MgCl_2_-30	b	0.02
MgCl_2_-90	0.05	MgSO_4_-70	b	0.02
CaCl_2_-90	0.05	CaCl_2_-90	b	0.02
CV%	50.08	CV%		27.14
*S.F.W.:*	n.s.	*S.D.W.:*		***

p ≤ 0.05 *, p ≤ 0.01 **, p ≤ 0.001 ***, n.s. (not significant), CV, Coefficient of Variation value.

S.F.W., Seedling Fresh Weight; S.D.W., Seedling Dry Weight.

In contrast, the 90 mM calcium chloride and 90 mM magnesium chloride treatments (both 0.05 g) resulted in the most pronounced reductions in seedling fresh weight, highlighting the suppressive effect of high salt concentrations on water retention and turgor-related growth processes. Moreover, a highly significant difference in seedling dry weight was observed among the treatments (p ≤ 0.001;*). The highest dry weight was recorded in the 30 mM magnesium sulfate treatment, which was not statistically different from the control group. This suggests that this particular treatment mitigated the adverse effects of salinity on biomass accumulation. Similarly, the 60 mM magnesium sulfate treatment exhibited a dry weight trend comparable to that of the control. By contrast, the lowest dry weights were recorded in the 70 mM magnesium sulfate (0.02 g) and 90 mM calcium chloride (0.01 g) treatments, indicating that these doses significantly inhibited biomass formation. These results are consistent with previous studies on *Lolium perenne*, where salt stress has been widely reported to reduce growth and biomass production (Rahimi et al., 2021; Yuan et al., 2021; Javaid et al., 2022; Tang et al., 2022).

Seedling water content is a critical physiological indicator of a plant’s response to salt stress. In this study, salt treatments had a statistically significant effect on seedling water content (p ≤ 0.05;*), indicating that different salt types and concentrations variably influenced the plant’s water retention capacity.

The highest water content (84%) was observed under the 70 mM magnesium sulfate treatment, which was found to be statistically higher than all other treatments (**Table 4**). This finding suggests that this treatment may have enhanced osmotic adjustment and water retention in *Lolium perenne* seedlings under salt stress conditions.

**Table 4:** Means and significance levels of seedling water ratio (%) and seedling dry matter ratio (%).

Treatments	Seedling water ratio (%)		Treatments	Seedling dry matter ratio (%)	
MgSO_4_-70	a	84	MgSO_4_-60	a	40
CaCl_2_-60	ab	80	Control	ab	30
Na_2_SO_4_-60	ab	79	MgCl_2_-70	ab	30
Na_2_SO_4_-40	ab	77	MgCl_2_-90	ab	30
CaCl_2_-70	ab	77	MgSO_4_-40	ab	30
MgCl_2_-30	ab	76	CaCl_2_-30	ab	27
Na_2_SO_4_-30	ab	76	CaCl_2_-40	ab	27
CaCl_2_-90	ab	75	MgCl_2_-40	ab	27
MgSO_4_-90	ab	75	MgCl_2_-60	ab	27
CaCl_2_-40	ab	75	MgSO_4_-30	ab	27
MgCl_2_-40	ab	74	Na_2_SO_4_-70	ab	27
CaCl_2_-30	ab	73	CaCl_2_-70	ab	23
MgSO_4_-30	ab	73	CaCl_2_-90	ab	23
MgCl_2_-60	ab	72	MgSO_4_-90	ab	23
MgCl_2_-70	ab	72	Na_2_SO_4_-30	ab	23
MgCl_2_-90	ab	71	Na_2_SO_4_-40	ab	23
Control	ab	70	Na_2_SO_4_-60	b	20
Na_2_SO_4_-70	ab	70	CaCl_2_-60	b	20
MgSO_4_-40	ab	69	MgCl_2_-30	b	20
MgSO_4_-60	b	63	MgSO_4_-70	b	17
CV%	7.75		CV%	24.64	
S.W.R.	*		S.D.M.	*	

p ≤ 0.05 *, p ≤ 0.01 **, p ≤ 0.001 ***, n.s. (not significant), CV, Coefficient of Variation value. S.W.R., Seedling Water Ratio; S.D.M., Seedling Dry Matter.

Relatively high-water content values were also recorded in the 60 mM calcium chloride (80%) and 60 mM sodium sulfate (79%) treatments; however, there was no statistically significant difference between these two treatments. This indicates that certain moderate salt concentrations may partially support water conservation, though the effect appears to be dependent on the specific salt compound used.

The control group and the 70 mM sodium sulfate treatment both exhibited water content values of 70%, which, although relatively low, were statistically similar to some other treatments. This result highlights that water content alone may not fully reflect the entire spectrum of physiological responses induced by salt applications.

The lowest water content (63%) was recorded in the 60 mM magnesium sulfate treatment, which was identified as the most limiting condition in terms of water retention capacity (**Table 4**). Additionally, seedling dry matter composition was analyzed. The results reflect the physiological impact of salt stress on plant development and are presented in **Table 4** as complementary parameters.

The 60 mM magnesium sulfate treatment exhibited the highest dry matter content at 40%, indicating a potentially beneficial effect on seedling development. The control group, as well as the 70 mM and 90 mM magnesium chloride treatments, yielded similar results, each with a dry matter content of 30%.

In contrast, the 70 mM magnesium sulfate treatment (17%), along with 60 mM calcium chloride, 60 mM sodium sulfate, and another 60 mM calcium chloride treatment (each 20%), exhibited the lowest dry matter content. These findings suggest that these specific treatments had detrimental effects on dry matter accumulation in the seedlings. Overall, the changes in water content did not always correspond directly with dry matter accumulation, indicating that these two parameters reflect different aspects of the plant’s physiological response to salinity The results obtained in this study are largely consistent with previous literature evaluating the effects of salt treatments on growth in *Lolium perenne* (Yuan et al., 2021; Ahmadi et al., 2023).

## 4 Conclusion

The expression levels of salt-responsive genes (*APX, GR, HMA* and *PCS*) in *Lolium perenne* were analyzed using qPCR following the application of various chemical salt compounds. According to one-way ANOVA results, *HMA* and *PCS* genes exhibited statistically significant expression differences compared to the control group. Under severe salt stress conditions—such as 90 mM CaCl_2_, 60 mM MgSO_4_, and 70 mM MgSO_4_—these stress-related genes were markedly upregulated.

Despite this enhanced genetic response, key growth parameters were negatively affected, indicating that gene induction alone is insufficient to fully mitigate the physiological consequences of salt stress. This reveals a clear mismatch between molecular activation and phenotypic performance, indicating that increased gene expression does not necessarily translate into improved growth under salinity. In contrast, under milder or moderate salt treatments (e.g., 40 mM Na_2_SO_4_), gene expression levels remained relatively stable, and plant growth was better maintained. It is also important to note that Ca²^+^ and Mg²^+^ salts possess dual roles under stress conditions; while they contribute to salinity, they also serve as essential nutrients involved in membrane stabilization, ionic balance, and signaling pathways, which may partially explain the more moderate physiological responses observed at lower concentrations. Overall, salt stress posed considerable challenges to plant development, manifested by oxidative damage, reductions in biomass, and impairment of physiological functions. These findings emphasize the diverse effects of different salt compounds on seedling performance, revealing that each salt type influences plant growth and physiological processes in distinct ways. Future research is recommended to focus on developing salt-tolerant genotypes and cultivars, particularly in *Lolium perenne* and similar perennial grass species, to enhance resilience under saline conditions. In addition, future studies should adopt a more integrative approach incorporating molecular responses, physiological traits, ion balance, and antioxidant capacity rather than focusing solely on general assessments of salt tolerance.

## Data Availability

The original contributions presented in the study are included in the article/supplementary material. Further inquiries can be directed to the corresponding authors.
